# Multimodal Deep Learning and Visible-Light and Hyperspectral Imaging for Fruit Maturity Estimation

**DOI:** 10.3390/s21041288

**Published:** 2021-02-11

**Authors:** Cinmayii A. Garillos-Manliguez, John Y. Chiang

**Affiliations:** 1 Department of Computer Science and Engineering, National Sun Yat-sen University, Kaohsiung 804, Taiwan; cgmanliguez@up.edu.ph; 2 Department of Mathematics, Physics, and Computer Science, University of the Philippines Mindanao, Davao City 8000, Philippines; 3 Department of Healthcare Administration and Medical Informatics, Kaohsiung Medical University, Kaohsiung 804, Taiwan

**Keywords:** multimodality, deep learning, hyperspectral imaging, fruit maturity, classification

## Abstract

Fruit maturity is a critical factor in the supply chain, consumer preference, and agriculture industry. Most classification methods on fruit maturity identify only two classes: ripe and unripe, but this paper estimates six maturity stages of papaya fruit. Deep learning architectures have gained respect and brought breakthroughs in unimodal processing. This paper suggests a novel non-destructive and multimodal classification using deep convolutional neural networks that estimate fruit maturity by feature concatenation of data acquired from two imaging modes: visible-light and hyperspectral imaging systems. Morphological changes in the sample fruits can be easily measured with RGB images, while spectral signatures that provide high sensitivity and high correlation with the internal properties of fruits can be extracted from hyperspectral images with wavelength range in between 400 nm and 900 nm—factors that must be considered when building a model. This study further modified the architectures: AlexNet, VGG16, VGG19, ResNet50, ResNeXt50, MobileNet, and MobileNetV2 to utilize multimodal data cubes composed of RGB and hyperspectral data for sensitivity analyses. These multimodal variants can achieve up to 0.90 F1 scores and 1.45% top-2 error rate for the classification of six stages. Overall, taking advantage of multimodal input coupled with powerful deep convolutional neural network models can classify fruit maturity even at refined levels of six stages. This indicates that multimodal deep learning architectures and multimodal imaging have great potential for real-time in-field fruit maturity estimation that can help estimate optimal harvest time and other in-field industrial applications.

## 1. Introduction

As an abundant source of antioxidant nutrients, vitamins, minerals, and fiber, fruits play a crucial part in human health outcomes [1]. Papaya (*Carica papaya* L. ), one of the major tropical fruits worldwide, has excellent flavor, high nutritional content, many pharmacological properties, and growing commercial value [2]. Papaya fruit consumption provides antioxidant nutrients, B vitamins, potassium, magnesium, and fiber that help prevent digestive system disorders and heart diseases [3]. Recent research also indicates that consumption of papaya leaf juice can rapidly increase the production of platelets, which is beneficial to dengue patients [4].

Global papaya production has reached 13.58 million tons in 2018, where about 60% is produced in Asia and 38% from other regions. Countries in Central America and the Caribbean are the top exporters of papaya with an estimated 219,400 tons or 77% of the world gross export followed by South America and Asia. Due to the high nutritional properties, papaya consumption has increased in many countries especially in North America and Europe with a total net import of 203,000 tons and 43,800 tons, respectively [5].

The tropical fruit industry in the Philippines and Taiwan contributes to the biggest source of earnings among food exports. In 2011, the Food and Agriculture Organization (FAO) reported that edible fruits in the Philippines are valued at US$940 million [6] and in 2018, the production of papaya, mango, and pineapple was about 164,300 tons, 676,900 tons, and 2,717,400 tons, respectively [5]. The export value of papaya, pineapple, and mango in Taiwan has increased to US$440,000, US$2.34 million, and US$13.34 million in the same year, respectively [7]. The high economic value of these fresh produce has led to different research studies on fruit cultivation, production, pre-harvest, and post-harvest handling, and fruit grading [6,8,9]. 

Post-harvest regulatory measures and quality standards on fresh produce imposed by both exporting and importing countries help reduce the risk associated with chemical safety and health to consumers [10]. In the export industry, fruit grading is one of the most important post-harvest operations. Produce of highest quality grade remains in the main flow while those of second- and third-grade quality are sorted out and marketed in less-demanding outlets [11,12]. 

Fruit maturity evaluates the physiological development, that is when the fruit continues to ripen even after harvesting. It directly affects the important quality characteristics such as the color, size, texture, flavor, and nutrient content, and hence the grade. Perishability and susceptibility to adverse handling and storage conditions are also dependent on harvest maturity [13]. Harvesting an under-mature fruit may reduce the taste quality upon ripening, while harvesting over-mature fruit can result in overripening during transportation for export [14]. For example, an overripened papaya or mango becomes soft and prone to mechanical damages that may substantially reduce its quality and increase its susceptibility to decay and microbial growth, and thereby affect food safety and consumer preference [15,16]. Thus, fruit maturity is a critical factor to preserve post-harvest life [12], reduce waste along the supply chain, and increase fruit quality [11]. More than 40% of food losses occur at post-harvest and processing levels in developing countries, while more than 40% of food losses in industrialized countries occur at retail and consumer levels. The estimated post-harvest losses of major food crops in the Philippines is about 27–42%, which is commonly caused by inaccurate maturity estimation, mechanical damage, weight loss, disease, and rotting [17]. These losses along with the increasing cost of sourcing skilled farm laborers in the agriculture industry may leave small profit margins [18,19]. 

Efficient assessment of fruit maturity and grade is indispensable for alleviating these challenges. Well-trained personnel with experience must classify fruit produce based on maturity and grade, although inspection results differ from one operator to another [20]. Destructive and non-destructive methods are carefully studied and meticulously performed to quantify important quality attributes such as the size, shape, texture, firmness, peel color, chlorophyll concentration, total soluble solids content (TSS), starch, and titratable acid [11]. For instance, juice extraction in a laboratory setup is necessary for a refractometer to measure the TSS or sweetness of papaya samples [21]. Traditional laboratory processes like the one mentioned are destructive and time-consuming, however recently promising non-invasive alternatives are proposed using computer vision techniques, machine learning, and imaging instruments e.g., visible-light or color imaging and hyperspectral imaging. Simultaneous assessment with both non-destructive and destructive laboratory measurements is still time-consuming and so the use of the non-destructive method alone is becoming promising [11]. Moreover, non-invasive early assessment of fruit maturity helps prevent early harvesting by estimating the optimal time of harvest. Given this high accuracy, the post-harvest losses due to the early harvest of under-mature fruit can be significantly reduced [14].

Hyperspectral and visible imaging coupled with the powerful capabilities of computer vision and deep learning are among the non-destructive systems used in smart and precision agriculture [22,23]. Hyperspectral imaging can generate hypercubes with high dimensionality that makes computation challenging. The spectral signatures extracted from hypercubes provide a high correlation with the internal properties of fruits, vegetables, and other materials. But with artificial intelligence, particularly deep learning which can analyze a large amount of data, more sensitive analyses on this dense spectral information can be achieved with higher performance.

Deep learning is the main driving force for breakthroughs in processing single modalities, e.g., images, video, audio, text, and speech. Using a general-purpose learning method like a convolutional neural network (CNN), it can independently acquire features from provided data with very minimal human engineering. This empowers it to perform greatly in tasks like object recognition, segmentation, and detection [24]. 

Recently, the role of deep learning in non-destructive methods of agriculture-related tasks is becoming increasingly important especially with the rise of smart and precision agriculture. Deep learning is highly explored today for its potential in various agricultural applications such as fruit detection and classification [25], fruit maturity estimation [26], plant disease detection [27,28,29], and early bruise or damage detection [15,30]. This approach can provide higher and more precise evaluation results compared to traditional and manual techniques. This qualifies it for utilization in important tasks in agriculture. 

Multimodal deep learning is another emerging approach that needs cultivation in terms of research. It works its way into applications from diverse research fields such as medical diagnostic methods [31], breast cancer prognosis prediction [32], activity and context detection [33], music genre classification [34], microblog sentiment prediction [35], malware detection [36], software program flaw detection [37], mobile traffic classification [38], and fruit detection [25]. This technique takes advantage of the information from one modality that complements and/or reinforces another. Recent research studies explored multimodal input datasets to overcome the limitations and complement the deficiencies of one modality, to help refine the weights, and to improve the accuracy or F1 score of a deep learning algorithm [25,39,40]. Experiments show that aggregating learned representations from different modalities help improve classification accuracy, which could be due to the complementary information embedded in different modalities used in the dataset [34]. For instance, a multimodal input composed of Red-Green-Blue or RGB, infrared, and depth images are loaded to the deep learning network to generate local features and global descriptors of the localized area for an application that helps visually impaired people. The findings showed that the lack of depth and sensitivity of an RGB image is supplemented by the information from the other input modalities i.e., infrared and depth images [39]. Furthermore, in a fruit detection study [25], the experimental results of utilizing RGB and near-infrared (NIR) images for detection of bell peppers demonstrated how multimodality improved detection, where the F1 score of 0.838 is higher than those with a single modality.

Fruit grading or fruit maturity estimation is an agricultural application that benefits from the potentials of multimodal deep learning. Besides the fruit hyperspectral signatures, interferences that samples possess (e.g., stem) and other morphological changes in the samples (e.g., size, shape, etc.,) must be considered when building a model. The use of visible imaging (i.e., RGB images) for fruit and vegetable quality assessment is widely used and studied [41]. With RGB images, morphological changes like the size, shape, texture, and irregularity in sample fruits can be precisely measured using computer vision techniques and machine learning as classifiers [42,43,44]. Utilizing both sensitivity of hyperspectral signatures and precision of RGB images can leverage the performance of a deep learning algorithm for estimation or classification tasks in fruit maturity or grading.

This exploration study features the first time deep learning architectures are evaluated for multimodal deep learning under papaya fruit maturity data. To the best of our knowledge, this study is the first one to combine two modalities: RGB images and hyperspectral data cube for papaya fruit maturity estimation. Furthermore, the convolutional and other processing layers constructed also represent an entirely different architecture.

The main objective of this study is to propose a multimodal deep learning model for high-performance fruit maturity estimation using hyperspectral and visible-light imaging technologies. Key contributions of this research are: (1) A database of hyperspectral reflectance and visible-light or RGB images of papaya fruit samples taken in a laboratory setup and classified into six maturity stages; (2) a systematic study of multimodal deep learning architectures applied to an agriculture-related task, specifically to papaya fruit maturity estimation, evaluated in terms of accuracy, top-2 accuracy, precision, recall, and F1-score; and (3) recommend a deep convolutional neural network that learns features over multiple modalities, specifically, a hyperspectral reflectance data cube and an RGB image. This strengthens the foundation of deep learning in agricultural applications such as real-time in-field fruit maturity estimation that can help estimate optimal harvest time. 

The rest of the paper contains the following sections: Section 2 Materials and Methods, Section 3 and Section 4 Results and Discussion, and Section 5 Conclusions.

## 2. Materials and Methods

### 2.1. Experimental Design

**Figure 1:**
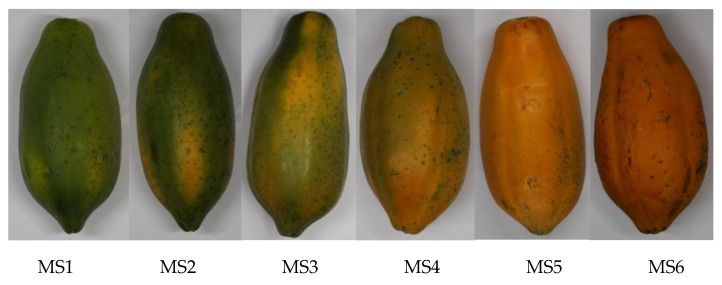
Six maturity stages of papaya fruit.

Maturity stages of papaya fruit samples are defined based on the visual characteristics of the peel color as illustrated in Table 1 (summary of papaya stages, description, number of RGB images, and hyperspectral (HS) data cubes) and Figure 1. Two hundred fifty-three (253) papaya fruits were purchased from a retail market in Kaohsiung, Taiwan. At least eight RGB images (one from each side: front, back, left, right; diagonal orientation of each side) and at least two hyperspectral images (two to four sides) per sample were acquired. All samples were classified into six maturity stages: green with a trace of yellow (MS1), more green than yellow (MS2), mix of green and yellow (MS3), more yellow than green (MS4), fully ripe (MS5), and overripe (MS6). We use six maturity stages same as [45] to test the sensitivity of the imaging and the deep learning model. The ground truth of the sample’s maturity stage is based on expert classification that is the same as [45] and compliant with the Philippine National Standard (PNS/BAFPS 33:2005). The differences in the number of samples from each maturity stage are due to their initial visual classification upon collection [46]. Measurements and images were gathered upon purchasing of samples (Day 1), Day 3, Day 5, and/or Day 7 of maturity. The fruits were stored in a controlled environment to even ripening. Out of the 253 papaya fruit samples, the numbers of RGB images and HS data cubes obtained are (520, 64), (570, 74), (964, 88), (707, 80), (749, 101), and (917, 105) for MS1 to MS6, respectively. A total of 4427 RGB images and 512 HS images were obtained. In terms of sampling, only at most 122 RGB images were collected in [25,46] which used machine learning methods: Faster R-CNN and random forest, respectively, while 15,000 images were acquired in [47], which also used Faster R-CNN. [30,48,49] collected and used 557, 300, and 240 HSI images for classification using deep learning methods: ResNeXt, GAN, and AlexNet, respectively. Thus, the number of samples, RGB and HS images gathered in this study, is by far among the highest based on recently published studies.

### 2.2. Visible-Light Image (VLI) Acquisition and Preprocessing

**Figure 2:**
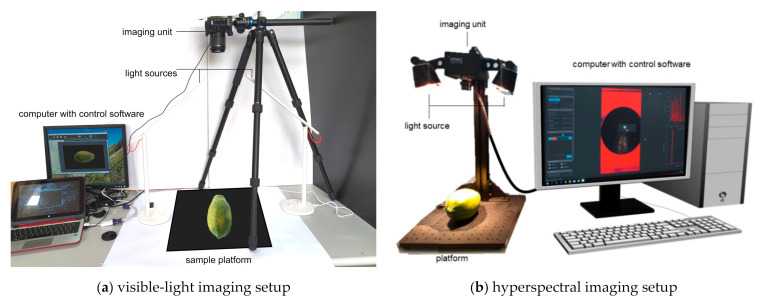
Experimental setups for image data acquisition using (**a**) visible-light and (**b**) hyperspectral imaging systems.

In the visible-light or visible imaging, the camera specifications used in VLI setting are Canon EOS 100D with 18.5 megapixels resolution, 5184 × 3456 pixels image size, and DIGIC 5 image processor. In the VLI setup shown in Figure 2a, the camera is positioned perpendicularly above the fruit sample using a tripod and is connected to a computer for remote capturing of images. Light sources in a laboratory setup are added to help illuminate the object and eliminate the shadows.

**Figure 3:**
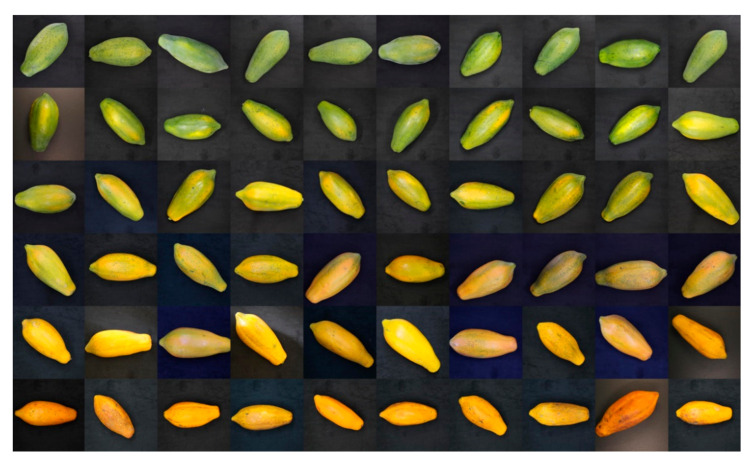
Example RGB images of papaya fruits acquired using visible-light imaging. Each row shows ten RGB images of papaya fruits that belong to each of the six maturity stages: MS1 in the first row, MS2 in the second row and so on until MS6 or overripe fruits in the last row.

For each papaya fruit sample, two RGB images are acquired for each side: one in a horizontal position and another in a diagonal position. The number of sides of the fruit depends on the angle where the fruit can lay flat on the imaging stage to obtain as many images as possible that helped increase the training dataset. From the original resolution, the images were cropped at the center to retain only the fruit sample in the middle of the image and then resized to 32 × 32 pixels due to computational limitation. Figure 3 shows examples of the preprocessed RGB images captured using VLI setup and stored in the database.

### 2.3. Hyperspectral Image (HSI) Acquisition and Preprocessing

**Figure 4:**
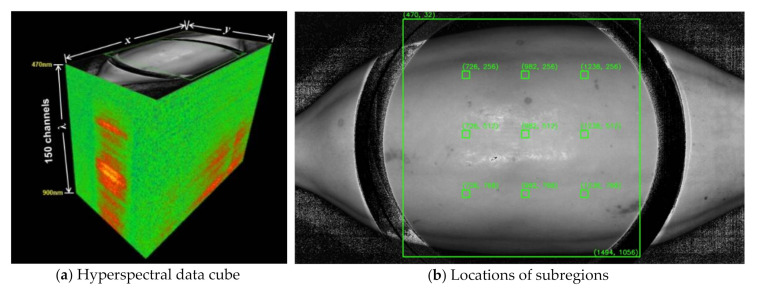
HSI data preprocessing to reduce dimensionality and increase dataset. A three-dimensional visualization of the raw spectral data, called a hyperspectral data cube, of a sample papaya is shown in (**a**). For the subregion extraction, (**b**) shows the locations of the nine 32 × 32 × 150 subregions obtained from the raw data cube.

An Imec SNAPSCAN visible/near-infrared (VNIR) hyperspectral imaging system is used in this study with 150 channels in between 470 nm and 900 nm wavelength data range and a spatial resolution of 2048 × 1088 pixels. The hyperspectral imaging system **,** as shown in Figure 4b, consists of the following parts: an imaging unit with active cooling system, a halogen illumination source, a platform, and a computer with corresponding control software. This imaging system uses linescan sensor technology to acquire HS reflectance data of an object through a push-broom scanning approach, in which acquisition is carried out line by line using the *x* and λ dimensions of the cube (Figure 4a).

**Figure 5:**
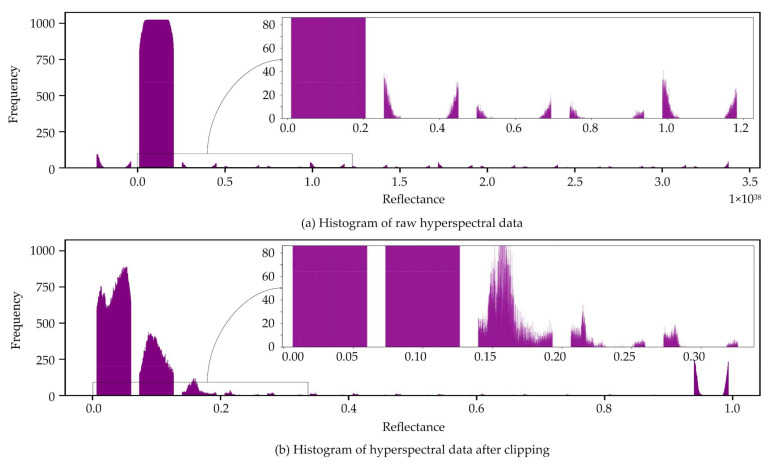
Histograms of a sample band in HS images.

The region of interest (ROI) in the hyperspectral image is the area corrected by the circular white reference. The spectral reflectance data enclosed by the 1024 × 1024 bounding box within this ROI in Figure 4b is further preprocessed by removing the outliers. If the surface has good reflectance, then the values are closer to 1. However, there are instances that HS images contain extremely high spectral reflectance values e.g., up to 3.5 × 10^38^ from the histogram shown in Figure 5a, mainly because of how the bidirectional reflectance distribution function (BRDF) is affected by the scene and camera geometry [50]. To satisfy range constraints, reflectance values are clipped to 0 and 1, or values less than 0 are set to 0 while extreme values greater than 1 are set to 1. The histogram of a sample band from an HS image after clipping the reflectance values is shown in Figure 5b.

From the bounded 1024 × 1024 ROI of the VNIR HS image (Figure 3b), nine (9) equally spaced subregions were obtained to increase the sample size and reduce computing cost and memory allocation because the image file size is reduced from about 1.3 GB to 600 KB only. A non-probabilistic sampling was done during the selection of nine patches from the hyperspectral images. Based on [45], the color measurements were obtained on three locations: one on the center and two on opposite sides longitudinally; [51] obtained measurements on six locations: two on the center and four on opposite sides. Following the guidelines from [45,51] and an 8-connectivity inspired data collection to obtain the data on the center of the HS image, three more locations were added to have a total of nine locations: three in the middle section, three near the apex, and three near the peduncle. Each subregion has a spatial resolution of 32 × 32 and a spectral resolution of 150. The same spatial resolution is used in [30] to reduce computation cost. These subregion data cubes were stored in the dataset to increase the number of input images to the neural network and help reduce overfitting.

### 2.4. Multimodal Deep Learning Framework

After data preprocessing, the RGB and HS images were then prepared for multimodal data integration. One important preparation is feature concatenation or data fusion of the two input modalities. Once the multimodal image dataset is ready, it is then fed to a deep learning method for classification to obtain the maturity estimation of the fruit sample. This subsection explains the framework used in multimodal implementation for deep learning to accomplish fruit maturity estimation tasks, which includes (i) feature concatenation and (ii) the deep learning architectures.

#### 2.4.1. Feature Concatenation

In digital image processing, an RGB image $a\left[m,n,\;c\right]$ is divided into *M* columns, *N* rows, and *C* channels or colors, i.e., *C* = 3 for red, green, and blue channels in this case. The value assigned to the integer coordinates $\left[m_{i},n_{i}\right]$ with {*m_i_* = 0, 1, 2, …, *M* − 1} and {*n_i_* = 0, 1, 2, …, *N* − 1}, and at channel or color $c_{i}$ with $\left\{c_{i}=0,\;1,\;2\right\}$ is $a_{i}\left[\;m_{i},n_{i},\;c_{i}\right]$ [52].

In hyperspectral image processing, the reflectance data $h\left[\;x,\;y,\;\lambda \right]$ obtained from the imaging system are formatted as a three-dimensional (3D) array (*M × N × S*), where *M* and *N* are the spatial dimensions and *S* is the spectral dimension that indicates the number of spectral wavelengths or bands used during data acquisition stage. This 3D array $h\left[\;x,\;y,\;\lambda \right]$ containing a set of two-dimensional images $h\left[\;x,\;y\right]$ obtained at varying wavelengths {λ = λ_0_, λ_1_, λ_2_, …, λ_*S*−1_} is also called a hyperspectral data cube.

In multimodal data integration, early fusion of data, also called as feature concatenation, is implemented at the input layer. In this approach, the imaging modalities are concatenated into a single multimodal input, RGB+HS data cube with final spatial dimensions of (*M × N*), and a total number of channels *G = C* + *S*, which generates a single yet large cross-modality input space. Each RGB+HS data cube in the dataset has 32 × 32 × 153 dimension and can now be fed to the deep learning model, specifically the convolutional neural network for training, validation, and testing. Table 2 shows the total number of RGB+HS multimodal data cubes for each maturity stage, which sums up to 4608 data cubes. This straightforward design makes it easier to train the network and access the whole space of imaging modalities containing cross-modality information needed for feature representations within the network.

#### 2.4.2. Multimodal Deep Learning Architectures

**Figure 6:**
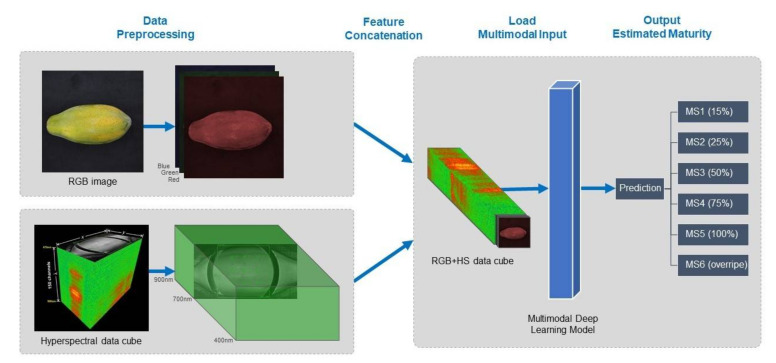
Multimodal deep learning framework for fruit maturity estimation.

Deep learning architecture plays an important role in multimodal tasks because it has significant properties advantageous to multimodal learning [33]. The representation-learning capabilities enable it to transform a raw multimodal input representation into a higher and slightly abstract one [24]. Specifically, the feature representation learning in this architecture does not require rigorous preprocessing to extract domain-specific features from each modality [33]. Thus, before feature concatenation, each modality is just lightly preprocessed and after feature concatenation, the multimodal input required minimal alterations. Figure 6 shows the multimodal deep learning framework for fruit maturity estimation.

The multimodal (MD) framework for deep learning mentioned in this subsection is implemented on seven multimodal convolutional neural networks (MD-CNNs), namely: MD-AlexNet, MD-VGG16, MD-VGG19, MD-ResNet50, MD-ResNeXt50, MD-MobileNet, and MD-MobileNetV2. These state-of-the-art architectures utilize convolution and pooling operations in their hidden layers of varying depth, width, and cardinality.

**Figure 7:**
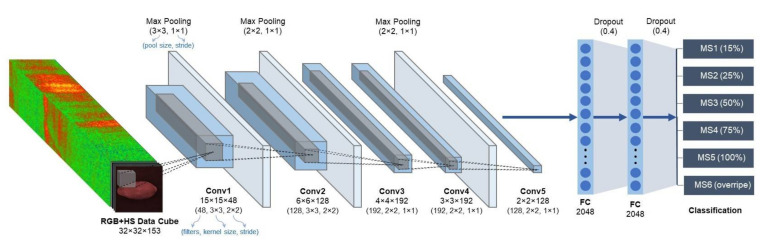
A multimodal deep learning architecture of AlexNet, a convolutional neural network with eight layers.

AlexNet together with the advent of GPUs is among those deep learning players that started the CNN revolution for object detection, and until today, it still has proven its robustness, flexibility, and competence despite its shallow depth [49]. AlexNet only consists of eight layers and that makes it train faster, which is ideal for real-time applications. Furthermore, other AlexNet features that inspire researchers to follow its lead in the trends in CNN are the use of ReLU Nonlinearity, exploitation of multiple GPUs, overlapping pooling, data augmentation, and dropouts [53]. Figure 7 shows an illustration of this MD-AlexNet architecture and other design parameters used in this study.

VGG16 and VGG19 are among those pioneering very deep convolutional networks. Both obtained state-of-the-art results in large-scale image recognition tasks. The very small convolution filters (3 × 3) allowed these networks to extend the depth to 16 and 19 weight layers. With the increase in depth comes an increase in representations too, which helped improve the network accuracy [54].

ResNet and ResNeXt are highly modularized networks giving emphasis to the importance of depth and cardinality of representations, respectively. ResNet stacks modules or blocks composed of a group of layers that further increased the depth, a characteristic inherited from VGG-nets, and addressed degradation problem (accuracy degrades rapidly as depth increases) by employing residual learning [55]. Its augment, ResNeXt, on the other hand, repeats building blocks that assemble a set of simple transformations having the same configuration. The implementation is like a split-transform-merge strategy, which results in a homogenous and multi-branch architecture setting with just a few hyperparameters. ResNeXt architectures introduced cardinality as a new dimension that essentially affects a network’s performance besides the depth and width [56]. 

MobileNet and MobileNetV2 are designed for mobile and embedded applications that take advantage of the CNN dominance in computer vision. The reduced computations and model size due to depth-wise separable convolutions (DSC) make MobileNet fit for applications under constrained environments (i.e., with limited resources). DSC applies factorization in the convolution operations, specifically, it splits the standard convolution into depth-wise convolution for filtering and pointwise convolution for combining. In this way, the computation cost is reduced substantially [57]. MobileNetV2 augments the former model on multiple tasks by employing a different technique called inverted residual structure. This sets the shortcut connections between the thin bottleneck layers and uses lightweight depth-wise convolutions for filtering, which allows memory efficient implementation—an important factor in mobile applications and embedded systems [58].

This study tested the capabilities of these MD-CNN models for the estimation of the six maturity stages of papaya fruits. The summary of the depth and the number of parameters used in each architecture during the actual implementation is shown in Table 3. Among the deep learning models used, MD-ResNet50 has the highest number of parameters while MD-AlexNet has the lowest with about 30.3 million and 4.9 million parameters, respectively.

These models are implemented using Python programming language, Anaconda, Tensorflow-GPU, Keras, and other packages for deep learning model development. They were trained with a learning rate of 0.0001 and batch size of 100 and deployed in a machine with Intel Core i5-9300H 2.40 GHz CPU (8 CPUs), NVIDIA GeForce GTX 1660Ti 6GB GPU, and 8 GB memory space running on Windows 10 Home 64-bit (10.0, Build 18362) system. The default values of the deep learning model development tools were used in these models except that of MD-AlexNet, which is shown in Figure 7. The dataset distribution used in all seven MD-CNN architectures is 2741 RGB+HS data cubes (60%) for the training set, 1383 (30%) for the validation set, and 484 (10%) for testing set, with a total of 4608 multimodal input data cubes. 

### 2.5. Performance Evaluation

The MD-CNN models are evaluated using accuracy, precision, recall, F1 score, and top-2 error rate. Accuracy is the proportion of correctly classified samples among the total number of samples examined and is computed as $Accuracy\;=\;\frac{\mathrm{TP}+\mathrm{TN}}{\mathrm{TP}+\mathrm{FP}+\mathrm{FN}+\mathrm{TN}}$, where TP is the number of true positives or correct classifications (positive class for binary classification (BC)), TN is the number of true negatives or correct classifications of the other class/es (negative class for BC), FP is the number of false positives or incorrect classifications (true negative predicted as positive in BC), and FN is the number of false negatives or missed classification (true positive predicted as negative in BC). This metric provides sound judgment for classification tasks with a balanced number of test samples per class.

Precision is the fraction of correct predictions out of all positive predictions whether correct or incorrect, which is calculated as $Precision\;=\;\frac{\mathrm{TP}}{\mathrm{TP}+\mathrm{FP}}$. While precision provides us with a quantitative measure of how exact the classifier’s prediction is, recall helps avoid waste by telling us how many samples per class can the classifier recognize accurately. Recall is the proportion of correctly detected out of all true/actual samples in a class, specifically it is computed as $Recall\;=\;\frac{\mathrm{TP}}{\mathrm{TP}+\mathrm{FN}}$.

F1-score is the harmonic mean recall (*R*) and precision (*P*): $F_{1}\left(R,\;P\right)=\frac{2RP}{R+P}$. This is a very valuable evaluation metric in this agricultural application because its assessment reflects the balance between the classifier’s precision and recall. In the fruit production and export industry, obtaining as many positives as possible makes a difference in the cost and throughput, and thus recall should be highly considered [30]. However, it is also worth noting that the industry values precision that assures them of good and exact prediction because these products should undergo a food safety and quality checking.

Top-2 error rate is the percentage of test samples where the actual/true classification is not among the two classifications or labels with the highest probabilities as predicted by the classifier. This is similar to the customary metrics used in ImageNet, which are top-1 and top-5 error rates. Since there is only a slight change in characteristics between adjacent maturity stages, human sorters may tend to classify the fruit on either one stage lower or higher than its correct maturity stage. Furthermore, given that this study involved experimentation, determining the models’ potential through obtaining the top-2 error rate is worth investigating in this study. This will better appreciate the performance of the multimodal deep learning methods in the agricultural field.

## 3. Results

In this paper, we explored multimodal deep learning architectures and examined the performance of the classification models, or simply classifiers, for estimating papaya fruit maturity stages. Specifically, in this section (1) we inspect the preliminary training results, (2) we examine the training and validation performance of the MD-CNNs, and (3) we compare the testing results of these classifiers.

### 3.1. Inspection of Preliminary Performance

**Figure 8:**
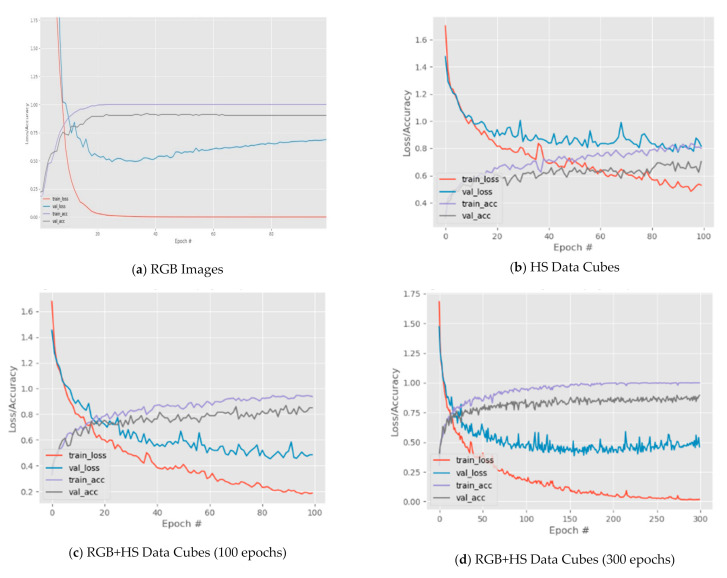
Preliminary training performance results of a 2L CNN using (**a**) RGB and (**b**) HS images, and a 2L MD-CNN using RGB+HS data cubes for (**c**) 100 and (**d**) 300 epochs.

Prior to the formal execution of the seven MD-CNNs, we carried out a preliminary experiment on a simple deep CNN with two hidden layers (2L CNN) using RGB images (Figure 8a) and HS data cubes (Figure 8b), and on a simple multimodal deep CNN with two hidden layers (2L MD-CNN) with the following configurations: 64 filters, 3 × 3 kernel size, 1 × 1 stride, ReLU as the activation function, categorical cross-entropy for the loss function, and Adam as optimizer using RGB+HS data cubes (Figure 8c) as input datasets. Figure 8a shows a fast and smooth convergence of the training loss and accuracy of the 2LCNN using only RGB images, which stabilized at about 40 epochs. Observing the validation loss and accuracy, there is still a need for improvement since the validation accuracy remains at about 90% while the validation loss increased from about 0.50 to 0.70 instead of decreasing or stabilizing. The graphs in Figure 8c,d show the behaviors of the 2L MD-CNN in terms of training and validation accuracy and loss for 100 and 300 epochs, respectively, and a learning rate of 0.001 and a batch size of 100. Given the same parameter settings, Figure 8c shows that with RGB+HS data cubes, the training and validation loss values converged faster to zero than with HS data cubes only as shown in Figure 8b. Inspecting the performance graphs deeper, we found that from epoch 20 to 40 the training and validation loss reduced by approximately 0.20 with RGB+HS data cubes as compared to that with HS data cubes which is only about 0.1. The lowest loss value in RGB+HS is about 0.20 while in HS it is about 0.50. Furthermore, in terms of accuracy, the highest training and validation accuracy reached in Figure 8b is approximately 85% and 70%, respectively. In Figure 8c, on the other hand, the highest training accuracy reached as high as nearly 95% and its highest validation accuracy is nearly 90%. Increasing the number of epochs up to 300 shows that the training loss converged to the value zero and the training accuracy reached 100% as presented in Figure 8d. Although, the validation loss remained between 0.40 and 0.60 range while the validation accuracy ranges between 85% and 90%.

### 3.2. Fruit Maturity Estimation Performance Comparison of MD-CNNs

The results on the training and validation sets are summarized in Table 4. All seven MD-CNNs obtained the best performance in terms of top-2 error rates and accuracy in the training set which are almost zero percent and 100%, respectively. In the validation set, however, the top three networks that retained their spots are MD-AlexNet, MD-VGG16, and MD-VGG19. Although there is a big difference in their depths, the first two MD-CNNs are at par in performance with the same top-2 error rates of 0.83%, and accuracy of 88.22% and 88.64%, respectively.

The comparison of results on the multimodal test set is presented in Table 5. The trend seen in the training and validation results is also reflected in the estimations of each MD-CNN on the multimodal test set. MD-VGG16, MD-AlexNet, and MD-VGG19 still obtained the most promising results among the seven MD-CNNs with F1-scores and top-2 error rates of (0.90, 1.45%), (0.88, 1.81%), and (0.87, 2.31%), respectively. The same pattern is also observed in terms of precision and recall. MD-VGG16 outperformed the rest in terms of the mentioned performance metrics. Interestingly, MD-AlexNet exhibited a comparable performance with MD-VGG19 by a difference of only (0.01, 0.50%), considering that only the former has a depth of 8 layers with almost 4.9 M parameters while the latter has 19 layers and 23.2 M. Similar to [49], despite being a small network, MD-AlexNet provided an accuracy of 98.6% in real-time strawberry ripeness (two ripeness stages only) estimation system and demonstrated its competitive advantage through dropouts [53].

**Figure 9:**
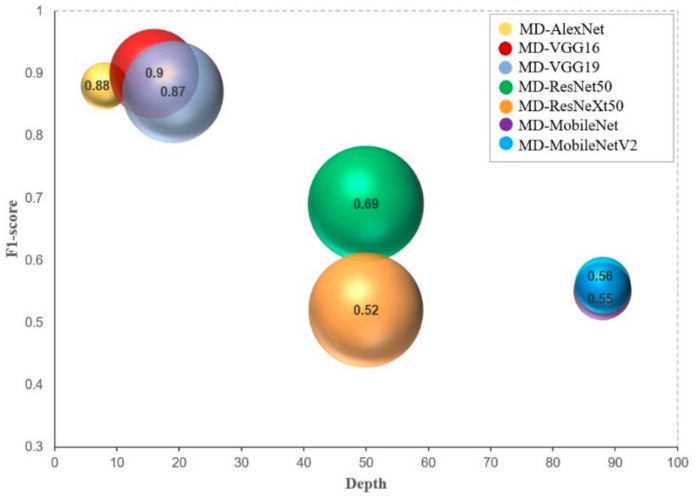
Bubble scatter plot of F1-score, depth, and number of parameters. While the first two data series identify the (*x*, *y*) location, the third one determines the bubble diameter.

Given these results (Table 5) and the bubble scatter plot in Figure 9, the depth, width, and the number of parameters of these architectures may not necessarily have a direct nor an inverse effect on their performance (F1-score) in a multimodal dataset. A diverse group of networks obtained promising outcomes while the others gave inferior ones. Observe that the two largest neural networks, i.e., with large bubble sizes such as MD-ResNet50 (50 layers, 30.3 M parameters) and MD-ResNeXt50 (50, 29.8 M), obtained very poor performance particularly MD-ResNeXt50 which practically produced a flip coin probability (50% chance). Moreover, even with very deep but also very thin neural networks i.e., with small bubble sizes, which are MD-MobileNet (88, 7.4M) and MD-MobileNetV2 (88, 7.0M), the same worse performance was exhibited on the test dataset.

In terms of top-2 error rates, however, all MD-CNNs showed effectiveness in estimating the true maturity stage of the samples within the two most probable labels or maturity stages. Consistently, VGG16 still ranked highest with a top-2 error rate of 1.45%, followed by MD-AlexNet (1.80%), MD-VGG19 (2.31%), MD-ResNet50 (7.30%), and the rest with error rates as low as 16.34% up to 18.36%.

## 4. Discussion

The preliminary result in this study adheres to the findings of [25] where the CNN with early fused RGB and NIR obtained a higher F1-score than the one using the NIR images alone, and even with that of [25,26] in which the deep learning methods using only RGB images obtained better results than the early fused multimodal technique. However, looking at the validation loss and accuracy, there is still room for improvement, and it is very interesting to note that the results of the CNN with only RGB images and only HS data cubes improved when the RGB images and HS data cubes were concatenated. Hence, despite the high-density data contained in the HS data cube, features from an RGB image can still contribute to the improvement in performance. As mentioned in Section 1, RGB images can contain an object’s morphological characteristics that the network can easily recognize, which can supplement the information derived from spectral data. Furthermore, [46] also explored the significant differences of various color spaces on papaya fruit ripening with only three maturity stages. Their study showed that the normalized RGB channels obtained higher accuracy and the RGB color channels achieved satisfactory results. Yet, it is still interesting to know the effect of the fusion of the various color channels and the hyperspectral data on fruit maturity estimation.

Among the multimodal deep learning models used in this study, MD-VGG16, MD-AlexNet, and MD-VGG19 were the top-performing models. These findings correspond to the studies of [26], which used only RGB images of three maturity stages of papaya and transfer learning on the deep learning models, and to [49], which utilized the first three principal components of hyperspectral images of strawberries and a pretrained AlexNet. From reference [26,49], VGG19 and AlexNet displayed superior performance, respectively. Similar to [49], despite being a small network, AlexNet without dropout layers provided an accuracy of 98.6% in real-time strawberry ripeness (two ripeness stages only) estimation system. In this study, transfer learning was not implemented because the nature of input images, which is multimodal, is different from that of the available pretrained models. Also, AlexNet is employed with dropout layers as shown in Figure 7 because of the dense multimodal input data, i.e., fused RGB+HS, and it also demonstrated its competitive advantage through these dropouts [53].

Moreover, the accuracy of the multimodal deep learning models especially those very deep models with depths of 50 and 88 can still be improved. Additionally, overfitting might occur in some of the aforementioned models. One possible reason causing the overfitting is the size of the images which is small and the depth of the CNNs are too high leading to very tightly adjusted training variables. This results in a dilemma because increasing the image size will also adversely cause insufficient memory errors.

In application, farms and production sites can use the proposed technology in this paper in either in-field or in-laboratory setups. For in-field utilization, the multimodal imaging system can be assembled as portable equipment for real-time non-destructive data collection and image analysis. On the other hand, the in-laboratory implementation requires sampling of fruits from the field and transporting these samples to the laboratory for analysis.

## 5. Conclusions

Multimodal techniques, particularly with imaging systems and deep learning, in agricultural applications are very advantageous since abundant information is made available to achieve highly sensitive analyses. Despite the high dimensionality of multimodal input, deep learning capability reduces the complexity of extracting salient features and accelerates associations of these features through its representation-learning networks. The breakthroughs in deep learning have led to more advanced instruments that increased productivity in various industries including agriculture.

In this study, we developed multimodal variants of deep learning models for a novel non-destructive, refined fruit maturity stage classification. To build the database of multimodal input, hyperspectral imaging and visible-light imaging acquired the hyperspectral data cubes and RGB images of papaya fruit samples, respectively, at six maturity stages from unripe stage to overripe stage. In data preprocessing, we implemented subregion extraction on the hyperspectral images to reduce their spatial dimension and increase the samples in the dataset. Through feature concatenation, we fused the data cubes and images to produce multimodal RGB+hyperspectral data cubes. Then, the models utilized the multimodal input data cubes and exhibited very promising results in fruit maturity estimation with up to 0.90 F1-score and as low as 1.45% top-2 error rate. Among the seven architectures, MD-VGG16 obtained the best performance, while MD-AlexNet and MD-VGG19 showed comparable outcomes. Overall, taking advantage of multimodal input coupled with powerful deep CNN models can classify fruit maturity even at refined levels of six stages. The results of this study offer great potential for multimodal deep learning and multimodal imaging to estimate fruit maturity in real-time. This further strengthens the foundation of deep learning in agricultural applications such as real-time in-field fruit maturity estimation that can help estimate optimal harvest time.

## Figures and Tables

**Table 1:** Maturity stages and data collection information of papaya fruit samples.

Maturity Stage	Descriptions	Number of RGB Images	Number of HS Data Cubes
MS1	Green with trace of yellow (15% ripe)	520	64
MS2	More green than yellow (25% ripe)	570	74
MS3	Mix of green and yellow (50% ripe)	964	88
MS4	More yellow than green (75% ripe)	707	80
MS5	Fully ripe (100% ripe)	749	101
MS6	Overripe	917	105

**Table 2:** Number of multimodal data cube for each maturity stage after feature concatenation.

Maturity Stage	Number of RGB+HS Multimodal Data Cubes (32 × 32 × 153 image size)
MS1	576
MS2	666
MS3	792
MS4	720
MS5	909
MS6	945

**Table 3:** Depth and the number of parameters of the seven MD-CNNs.

Deep Learning Model	Depth	Number of Parameters
MD-AlexNet	8	4,938,982
MD-VGG16	16	17,956,038
MD-VGG19	19	23,265,734
MD-ResNet50	50	30,358,790
MD-ResNeXt50	50	29,819,206
MD-MobileNet	88	7,475,590
MD-MobileNetV2	88	7,028,998

**Table 4:** Training and validation performance comparison of the seven MD-CNNs in terms of accuracy and top-2 error rate using 2741 and 1383 RGB+HS data cubes for the training set and validation set, respectively.

Deep Learning Model	Training		Validation	
	Top-2 Error Rate (%)	Accuracy (%)	Top-2 Error Rate (%)	Accuracy (%)
MD-AlexNet	0.00	100.00	0.83	88.22
MD-VGG16	0.00	100.00	0.83	88.64
MD-VGG19	0.00	100.00	1.86	85.74
MD-ResNet50	0.00	99.34	7.44	66.53
MD-ResNeXt50	0.04	99.42	16.12	56.40
MD-MobileNet	0.04	99.27	16.32	56.40
MD-MobileNetV2	0.04	99.23	19.63	55.37

**Table 5:** Performance comparison of the seven MD-CNNs in terms of precision, recall, F1-score and top-2 error rate on multimodal test set.

Deep Learning Model	Precision	Recall	F1-Score	Top-2 Error Rate
MD-AlexNet	0.8850	0.8817	0.88	1.8077
MD-VGG16	0.9016	0.9033	0.90	1.4461
MD-VGG19	0.8733	0.8733	0.87	2.3138
MD-ResNet50	0.7516	0.6850	0.69	7.3030
MD-ResNeXt50	0.6150	0.5550	0.52	16.3413
MD-MobileNet	0.5617	0.5633	0.55	16.9197
MD-MobileNetV2	0.5783	0.5667	0.56	18.3659

## Data Availability

The data presented in this study are available on request from the corresponding author. The data will be publicly available when this paper is already published.
